# Tomato Root Growth Inhibition by Salinity and Cadmium is Mediated by S-Nitrosative Modifications of ROS Metabolic Enzymes Controlled by S-Nitrosoglutathione Reductase

**DOI:** 10.3390/biom9090393

**Published:** 2019-08-21

**Authors:** Tereza Jedelská, Veronika Šmotková Kraiczová, Lucie Berčíková, Lucie Činčalová, Lenka Luhová, Marek Petřivalský

**Affiliations:** 1 Department of Biochemistry, Faculty of Science, Palacký University, CZ-783 71 Olomouc, Czech Republic; 2 Present address: Department of Immunology, Faculty of Medicine and Dentistry, Palacký University, CZ-77900 Olomouc, Czech Republic; 3 Present address: Department of Environmental Protection Engineering, Faculty of Technology, Tomas Bata University in Zlín, 760 01 Zlín, Czech Republic

**Keywords:** abiotic stress, cadmium, nitric oxide, reactive oxygen species, root growth, S-nitrosation, S-nitrosoglutathione reductase, salinity, *Solanum habrochaites*, *Solanum lycopersicum*

## Abstract

S-nitrosoglutathione reductase (GSNOR) exerts crucial roles in the homeostasis of nitric oxide (NO) and reactive nitrogen species (RNS) in plant cells through indirect control of S-nitrosation, an important protein post-translational modification in signaling pathways of NO. Using cultivated and wild tomato species, we studied GSNOR function in interactions of key enzymes of reactive oxygen species (ROS) metabolism with RNS mediated by protein S-nitrosation during tomato root growth and responses to salinity and cadmium. Application of a GSNOR inhibitor N6022 increased both NO and S-nitrosothiol levels and stimulated root growth in both genotypes. Moreover, N6022 treatment, as well as S-nitrosoglutathione (GSNO) application, caused intensive S-nitrosation of important enzymes of ROS metabolism, NADPH oxidase (NADPHox) and ascorbate peroxidase (APX). Under abiotic stress, activities of APX and NADPHox were modulated by S-nitrosation. Increased production of H_2_O_2_ and subsequent oxidative stress were observed in wild *Solanum habrochaites*, together with increased GSNOR activity and reduced S-nitrosothiols. An opposite effect occurred in cultivated *S. lycopersicum*, where reduced GSNOR activity and intensive S-nitrosation resulted in reduced ROS levels by abiotic stress. These data suggest stress-triggered disruption of ROS homeostasis, mediated by modulation of RNS and S-nitrosation of NADPHox and APX, underlies tomato root growth inhibition by salinity and cadmium stress.

## 1. Introduction

Roots perform a number of essential functions during plant growth and development, such as water and mineral absorption, acting as a nutrient reservoir and anchoring the plant in the soil. Plants have evolved a plethora of complex mechanisms to regulate various physiological and pathological processes in roots and signaling pathways of plant hormones; cytokinins, auxins and ethylene have emerged as crucial players in these processes (reviewed in [1]). The key processes determining the root system architecture, including primary root elongation, adventitious and lateral root formation and root hair differentiation involve the participation of reactive nitrogen species (RNS), mainly nitric oxide (NO), and reactive oxygen species (ROS), such as superoxide and hydrogen peroxide (H_2_O_2_) [2,3]. Recent research has uncovered several regulatory NO-dependent post-translational modifications, such as cysteine S-nitrosation, or tyrosine nitration, showing that NO is able to regulate the metabolism and levels of ROS and RNS, allowing plants to fine-tune specific responses to different stimuli [4,5]. NADPH oxidase analogue D (rbohD) represents an important enzyme source of ROS production in the plant cell membrane. RbohD is known to be S-nitrosated during the plant hypersensitive response (HR) at Cys 890, which thereby reduces its activity and ROS accumulation. In the later stages of HR, this redox modification acts as a negative feedback mechanism limiting the extent of cell death due to decreased ROS production [4].

NO-dependent signaling has been proposed to control ROS levels by activating ROS scavenging enzymes, such as catalase and superoxide dismutase, during stress responses [6]. Antioxidant enzymes involved in the glutathione-ascorbate cycle, ascorbate-peroxidase (APX) in particular, were also identified as S-nitrosation targets in multiple plants like Arabidopsis, soybean, rice, potato, poplar and pea [7,8,9,10,11] and tobacco BY-2 cells [12]. Auxin treatment induces de-nitrosation and inhibition of APX1 activity in Arabidopsis roots and S-nitrosated APX1 shows higher activity compared to the denitrosylated form [13]. S-nitrosation of Cys32 positively regulates APX1 enzymatic activity and immune responses in Arabidopsis plants [14]. Moreover, pea cytosolic APX is regulated by NO-derived RNS: nitration of Tyr235 causes the deactivation of APX activity, whereas S-nitrosation of Cys32 restores its activity [15].

S-nitrosoglutathione reductase (GSNOR, EC 1.1.1.284) is recognized as a decisive component in the maintenance of intracellular levels of S-nitrosoglutathione (GSNO), and indirectly also of protein S-nitrosation and NO homeostasis in general. GSNOR catalyzes an NADH-dependent reduction of GSNO to oxidized glutathione (GSSG) and ammonium, considered as an irreversible deactivation of NO [16,17]. GSNOR activity is required for normal plant development and fertility under optimal growth conditions [18,19,20,21,22,23]. Modulations of GSNOR activity were observed during multiple types of abiotic stress conditions (reviewed in [24]). Long-term salinity stress led to increased GSNOR activity in pea isolated mitochondria [25]. The absence of GSNOR has been shown to render enhanced sensitivity to alkaline stress in tomato, which was proposed to result from excessive accumulation of NO and S-nitrosothiols during this stress [26]. GSNOR expression and activity were increased by an NO donor in aluminum-treated rice plants. Suppressing GSNOR enzymatic activity using specific GSNOR inhibitors increased Al-induced accumulation of RNS and aggravated damage to rice plants [27]. In *Arabidopsis thaliana*, NO derived from nitrate assimilation inhibits GSNOR through S-nitrosative modification(s) of protein cysteines, which results in decreased GSNO degradation and increased S-nitrosothiols levels [28]. Moreover, the S-nitrosothiol feedback loop can regulate nitrogen flux through the nitrite assimilation pathway and control its bioavailability in plant cells by modulating its own consumption.

Soil salinity is one of the key limiting factors for plant growth and development and represents an important threat for crop production worldwide. High salt concentrations generate an ionic imbalance, hyperosmotic stress and subsequent oxidative stress affecting germination, plant growth and survival [29]. Similarly, heavy metals toxicity in plants involve disturbances in the antioxidant defense and induction of oxidative stress, which results in strong growth inhibition, decreased transpiration, photosynthesis and chlorophyll content [30]. This work expands current knowledge about the role of ROS and RNS in plant development and responses to abiotic stress conditions with a focus on the regulatory role of S-nitrosation of enzymes involved in ROS metabolism. Two tomato genotypes, a cultivated genotype *Solanum lycopersicum* cv. Amateur and a wild genotype *Solanum habrochaites*, were used as model plants in continuation to previous studies on *Solanum* spp. focused on the role of ROS and RNS during pathogenesis [31,32,33,34]. *S*. *habrochaites* is native to high altitude habitats in the Andean mountains [35], and various accessions were used as a source for increased abiotic tolerance as well as resistance to fungal pathogens [35,36]. The previous study on biochemical and structural characterization of GSNOR from tomato [22] pointed out at a high inhibitory effect of N6022, a compound developed as a potent inhibitor of human GSNOR [37]. Here, the N6022 inhibitor was used for in vivo studies to investigate the GSNOR role in plant development and stress responses to uncover the involvement of RNS in the regulation of the key enzymes of ROS metabolism.

## 2. Materials and Methods

### 2.1. Chemicals

All chemicals were of analytical purity grade purchased from Merck-Sigma (Steinheim, Germany) or Bio-Rad (Hercules, USA). GSNO was synthesized by nitrosation of L-glutathione by sodium nitrite in HCl [38] and its quality was checked spectrophotometrically in comparison to a commercial GSNO sample (Calbiochem, San Diego, USA). N6022 was purchased from Axon Medchem (Groningen, Netherlands).

### 2.2. Plant Material

Plant seeds were sterilized for 30 s in 70% ethanol and then for 30 min in 3% solution of commercial bleach containing sodium hypochlorite. Seeds were washed 3 times with sterile water, transferred to Petri dishes containing three layers of sterile filter paper moistened with sterile water and kept for 3 days in the dark at 25 °C. Ten germinated seeds were sown in one row in square Petri dishes containing a solid MS agar medium with vitamins (Duchefa, cat. n. M0222, 4.3 g/L), 10 g/L sucrose and tested compounds: a GSNOR inhibitor N6022 in 0.1, 1 and 10 µM concentration alone or in combination with 100 µM NO donor GSNO or 100 µM NO scavenger 2-phenyl-4, 4, 5, 5,-tetramethylimidazoline-1-oxyl 3-oxide (PTIO). Responses to abiotic stress conditions were studied with growth media supplemented with 50, 100 or 150 mM NaCl or 50, 100 or 150 μM CdCl_2_. Then, dishes were placed for 9 days in a growth chamber with a 18/15 °C (day/night) temperature regime an 12 h photoperiod, the irradiance of 100 μE·m^−2^/s was provided by warm-white fluorescent lamps.

### 2.3. Root Sampling and Preparation of Root Extracts

Seedlings were removed from the agar media, the root part excised and the length of the primary roots and the fresh weight determined. Roots were then ground using a mortar and pestle in liquid nitrogen and an extraction buffer (50 mM Tris-HCl pH 7.5, 0.2% Triton X-100, 2 mM dithiothreitol and 1 mM phenylmethylsulfonyl fluoride) in 1:2 (*w*/*v*) ratio. Homogenates were centrifuged at 16,000× *g* for 30 min at 4 °C.

### 2.4. Measurement of S-Nitrosothiol Content

S-nitrosothiol content was determined by a modified Saville method [39]. Root extracts (5 μL) were incubated in 96-well microplates for 5 min with 100 μl of 3.5% sulphanilamide in 0.5 M HCl with or without 1% HgCl_2_, and then for additional 5 min with 100 μL of 0.1% N-(1-naphthyl)-ethylenediamine dihydrochloride in deionized water. Absorbance was read at 540 nm with a microplate reader (Synergy HT, BioTek Instruments, USA). S-nitrosothiols were quantified from absorbance differences measured with and without added HgCl_2_, using a calibration curve prepared with GSNO solutions. The results were calculated per milligram of total protein measured by the Bradford method using BSA as a calibration standard [40].

### 2.5. Measurement of APX and GSNOR Activity

Supernatants of root extracts were desalted using gel filtration columns (NAP-10, GE Healthcare, USA). APX) activity was assayed as the rate of ascorbate oxidation in the presence of H_2_O_2_. The reaction mixture comprised 1.75 mM ascorbate, 0.7 mM H_2_O_2_, 0.1 M potassium phosphate buffer (pH 7.0) and enzyme extract. The decrease in absorption at 290 nm was monitored for 5 min at 25 °C and the amount of oxidized ascorbate was calculated using the molar extinction coefficient of H_2_O_2_ ε_290_ = 2.8 mM^−1^·cm^−1^). Enzyme activity was expressed in μmol of oxidized ascorbate per minute and g of fresh weight.

GSNOR activity was assayed as described previously [22,41]. Plant extracts were incubated in a reaction mixture containing 20 mM Tris-HCl pH 8.0 and 200 µM NADH. The reaction was started with freshly prepared GSNO solution at final 400 µM concentration and the decrease in absorbance was monitored at 340 nm. The activity was expressed as 1 nmol of NADH consumed min^−1^ g^−1^ of fresh weight, using the molar extinction coefficient of NADH ε340 = 6.22 mM^−1^·cm^−1^.

### 2.6. Measurement of NADPH Oxidase Activity

Roots were homogenized in an extraction buffer containing 0.25 M sucrose, 50 mM HEPES (pH 7.2), 3 mM EDTA, 1 mM DTT, 3.6 mM L-cysteine, 0.1 mM MgCl_2_, 0.6% PVP and protease inhibitor cocktail (Complete, Roche). The homogenate was filtered through a nylon mesh and centrifuged at 10000 xg for 45 min at 4°C. The supernatant was centrifuged again at 203,000× *g* for 60 min at 4 °C and the pellet of microsomal membrane fraction was re-suspended in ice-cold 10 mM Tris-HCl, pH 7.4. The superoxide-generating activity of NADPH oxidase was measured by the XTT method [42]. The reaction mixture contained 50 mM Tris-HCl buffer (pH 7.5), 1 mM XTT and 1 mM NADPH. The reaction was initiated by addition of a membrane fraction sample containing 20 μg of protein. The rate of XTT reduction by O_2_^−^ was determined at 492 nm and the amount of produced O_2_^−^ calculated using the molar extinction coefficient ε_492_ = 21.6 mM^−1^·cm^−1^.

### 2.7. Quantification of Gene Expression by qPCR

Total RNA from 100 mg of root tissue was extracted using the NucleoSpin Plant RNA kit, including DNaseI digestion (Macherey-Nagel, Germany). cDNA was synthesized from 1 μg of total RNA using oligo(dT)15 primer and Transcriptor High Fidelity Reverse Transcriptase (Roche, Branchburg, USA). The reaction mixture was incubated at 42 °C for 30 min followed by heat inactivation of reverse transcriptase by incubation at 70 °C for 5 min. Real-time PCR was performed using an ABsolute SYBR Green ROX Kit (ABgene Limited, Epsom, UK) on a CFX96 Touch Real-Time PCR Detection System (Bio-Rad, USA). The SYBR Green signal was standardized with an internal passive reference dye (1 mM ROX) included in the SYBR Green PCR mix. Primer pairs used to amplify analysed genes are listed in Supplementary Table S1. The following program was applied: initial DNA polymerase activation 95 °C for 15 min, then 40 cycles at 95 °C for 15 s, 60 °C for 30 s and 72 °C for 30 s. The specificity of PCR amplification was checked with a melting curve program 55–95°C following the final cycle of PCR. PCR conditions were optimized for a high amplification efficiency >95% for each used primer pair and negative controls in the absence of template were also performed. Relative quantification of gene expression was normalized to housekeeping genes GAPDH and EF1using the Pfaffl′s method [43].

### 2.8. Detection of NO, ONOO^−^ and ROS

RNS and ROS detections were performed in the root apical parts (4 mm segments) using a fluorescence microscope. Root segments were incubated 20 min in 20 μM DAF-FM DA in 10 mM Tris-HCl buffer, pH 7.4 (NO detection), 30 min in 20 μM APF (3′-(p-aminophenyl) fluorescein) in 10 mM Tris-HCl buffer, pH 7.4 (ONOO- detection), or 10 min in 20 μM H_2_DCF DA in 10 mM Tris-HCl buffer, pH 7.4 (ROS detection), washed three times 5 min in the Tris-HCl buffer, transferred to a microscopic slide with a drop of 50% glycerol in 10 mM Tris-HCl buffer, pH 7.4, and covered with a coverslip. Fluorescence was subsequently detected using excitation at 460–490 nm and emission at 520 nm (BX50, Olympus, Japan). Root segments pre-incubated 10 min with 200 μM NO scavenger cPTIO, 20 μM ONOO^−^ scavenger ebselen or 20 mM ROS scavenger ascorbate served as negative controls.

### 2.9. Purification and Detection of S-Nitrosated Proteins

S-nitrosated proteins were analysed using the modified biotin-switch technique [44]. Briefly, frozen plant material was extracted in 1:2 (*w*/*v*) ratio with HEN buffer (100 mM HEPES-NaOH pH 7.4, 10 mM EDTA, 0.1 mM neocuproine) containing 1% (*v*/*v*) Triton X-100 and protease inhibitor cocktail (Roche). Cell debris was removed by centrifugation (16,000× *g*, 30 min, 4 °C) and protein concentration determined by Bradford assay with BSA as a calibration standard [40]. Samples were incubated with 20 mM methyl methanethiosulfonate and 2.5% SDS at 50 °C for 30 min with repeated vortexing to block non-nitrosylated free Cys thiols. Residual reagents were removed by protein acetone precipitation with three volumes of ice-cold acetone and precipitated proteins were re-suspended in 0.1 mL of HEN buffer containing 1% SDS per milligram of protein in the starting sample. Biotinylation was achieved by adding biotin-HPDP (Thermo Fisher-Scientific, Waltham, USA) and 1mM ascorbate with further incubation at room temperature for 1 h in the dark. After biotinylation, the proteins were precipitated with ice-cold acetone and subjected to affinity purification of biotinylated proteins by NeutrAvidine agarose (Thermo Fisher-Scientific) and to subsequent Western blot analysis as described previously [44]. Precipitated proteins were resuspended in HENS buffer (100 μL per mg of protein in the starting sample) and 2 volumes of the neutralization buffer (20 mM HEPES, pH 7.7, 100 mM NaCl, 1 mM EDTA, and 0.5% (*v*/*v*) Triton X-100). Biotinylated proteins were incubated for 1 h at room temperature with the NeutrAvidine agarose (30 μL per mg of protein). The agarose-matrix was washed with 20 volumes of the washing buffer (600 mM NaCl in the neutralization buffer) and bound proteins eluted with 100 mM β-mercaptoethanol in the elution buffer (20 mM HEPES, pH 7.7, 100 mM NaCl, 1 mM EDTA) and analyzed by SDS-PAGE, followed by immunoblotting using rabbit polyclonal anti-APX (dilution 1:1000) or anti-RBOHD (dilution 1:1000) antibodies (Agrisera, Sweden) as primary and alkaline phosphatase-conjugated goat anti-rabbit Ig antibodies (Merck-Sigma, USA) as secondary antibodies. For protein loading control in Western blot experiments, rabbit polyclonal anti-actin antibodies (Agrisera, Sweden) were used.

### 2.10. Quantification of Protein Nitration

Proteins in root extracts were separated by SDS-PAGE in 12% polyacrylamide gels, transferred to a PVDF membrane with a semi-dry Trans-Blot cell (Bio-Rad, Hercules, CA, USA) and probed with mouse monoclonal antibody against 3-nitrotyrosine (Sigma-Aldrich, St. Louis, USA), diluted 1:1000 [45]. For immunodetection, a goat anti-(mouse IgG)-horseradish peroxidase conjugate (diluted 1:10,000; Pierce, Thermo Fisher Scientific, Waltham, USA) was used after washing in TBS digital images were quantified using Image Studio software (LI-COR Biosciences, Lincoln, USA).

### 2.11. Image Analysis

The evaluation of fluorescence signal intensity in apical parts of the roots corresponding to NO, ONOO- or ROS was performed on obtained images by ImageJ software v1.33 (National Institute of Health, Bethesda, USA).

### 2.12. Statistical Analysis

Experiments were performed in triplicate for each *Solanum* spp. genotype and each experimental setting, and the results represent mean ± SD values obtained from at least three independent biological experiments. Statistical significant differences in values for treated samples compared to non-treated controls were assessed using one-way ANOVA.

## 3. Results

### 3.1. Differences in Parameters of ROS and RNS Metabolism in the Roots of Solanum spp. Genotypes Grown Under Non-Stress Conditions

To address the role of GSNOR and S-nitrosation in RNS and ROS-mediated signaling in plant root growth and responses to abiotic stress conditions, as a continuation to our previous studies [31,32,33,34,41], we studied two genotypes of *Solanum* spp.: A cultivated *S. lycopersicum* cv. Amateur, and a wild *S. habrochaites* f. glabratum genotype. To compare the developmental characteristics of selected genotypes cultivated in vitro on agar medium, in the initial part of the study we analyzed selected physiological and molecular parameters in the roots of 9-day seedlings (Supplementary Figure S1, Table S2). In accordance with previous reports on plants cultivated in the soil, wild *S. habrochaites* showed slower growth of hypocotyl and roots in agar media, determined also as decreased root fresh weight in comparison to *S. lycopersicum* cv. Amateur. Interestingly, except for peroxynitrite (ONOO^−^) levels, significant differences were found in various parameters of ROS/RNS levels and enzymes of their metabolism. Using fluorescence probes, the levels of NO were significantly higher in *S. lycopersicum*, whereas *S. habrochaites* roots showed slightly increased levels of ROS. Both S-nitrosothiol levels and GSNOR activity were approx. two times higher in *S. lycopersicum*; as well as the gene expression and activity of APX. Surprisingly, we observed a lower activity of *S. habrochaites* NADPH oxidase while its gene expression was higher compared to the other genotype.

### 3.2. Inhibition of GSNOR Stimulated Root Growth in both Solanum spp. Genotypes

**Figure 1:**
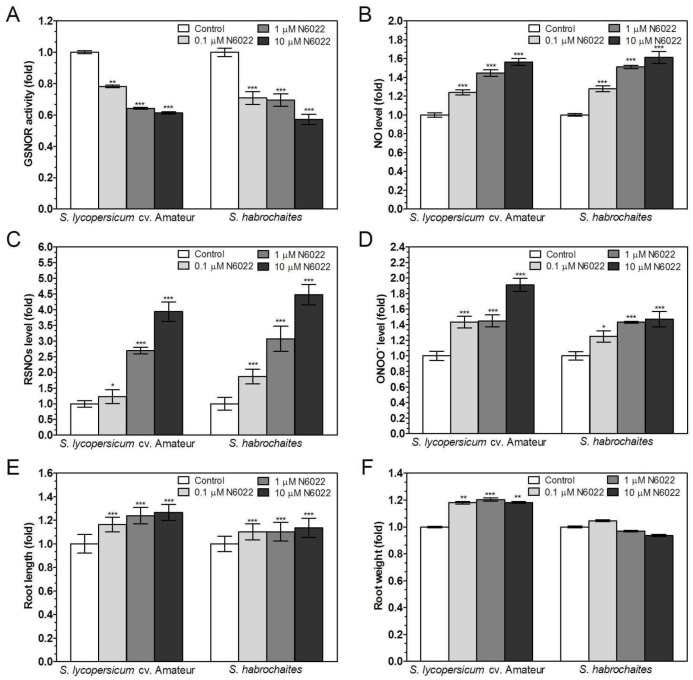
Effects of GSNOR inhibitor N6022 on GSNOR activity, RNS levels and root growth in two *Solanum* spp. genotypes. Plants were grown in the growth media containing 0, 0.1, 1 and 10 μM N6022. (**A**) GSNOR activity was evaluated spectrophotometrically at 25 °C by monitoring the decrease of NADH absorbance at 340 nm. (**B**) Changes in NO levels in apical parts of roots were monitored by confocal microscopy using 20 μM DAF-FM-DA. (**C**) The S-nitrosothiol content was determined by the modified Saville method at λ = 540 nm. (**D**) Changes in ONOO^−^ levels in apical parts of roots were monitored by confocal microscopy using 20 μM APF. The effect of N6022 was also studied on physiological parameters of (**E**) root length and (**F**) root fresh weight. The data are presented as means ± SD (A–D: *n* ≥ 3; E–F: *n* = 30) relative to the value of the tested parameter measured for control plants grown in media without N6022. Significantly different means from the control value are denoted by asterisks (ANOVA, * *p* < 0.05, ** *p* < 0.01, *** *p* < 0.001).

To elucidate the GSNOR role in the regulation of NO and RNS metabolism during plant root growth and responses to salinity and heavy metal stress in studied *Solanum* spp. genotypes, we employed a pharmacological approach using a non-competitive inhibitor of animal GSNOR N6022 [37], previously characterized as an efficient inhibitor of tomato GSNOR in vitro [22]. Decreased GSNOR activity and a concomitant strong increase in S-nitrosothiol levels and also induced NO and ONOO^−^ levels were detected with increasing concentrations of N6022 for both tomato genotypes (Figure 1). Similarly, GSNOR inhibitor induced increased NO levels whereas ONOO^−^ production was increased only in *S. habrochaites* grown in media containing 1 and 10 μM N6022 (Figure 1B,D). N6022 showed stimulatory effects on the root growth mainly in *S. lycopersicum* cv. Amateur; similarly, significant stimulating effects on the root weight were observed in this genotype only (Figure 1E,F).

### 3.3. Effects of GSNOR Inhibition on Plant Root Growth are Mediated by Modulation of NO and S-Nitrosothiol Levels

**Figure 2:**
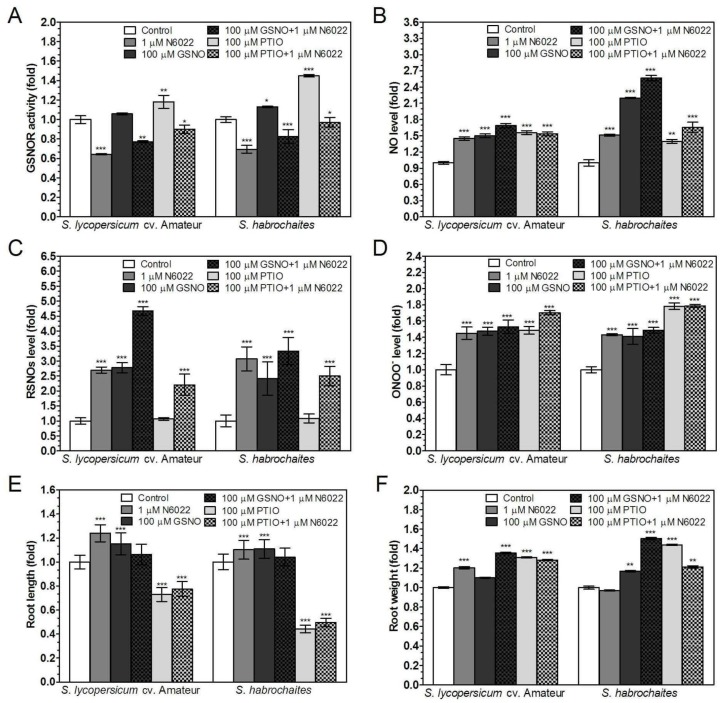
Effects of RNS modulators on GSNOR activity, RNS levels and root growth in two *Solanum* spp. genotypes. Plants were grown in the growth media supplemented with one of selected RNS modulators: 1 μM N6022 (GSNOR inhibitor), 100 μM GSNO (S-nitrosation reagent), 100 μM PTIO (NO scavenger). (**A**) GSNOR activity was evaluated spectrophotometrically at 25 °C by monitoring the decrease of NADH absorbance at 340 nm. (**B**) Changes of NO levels in the root apical parts were monitored by confocal microscopy using 20 μM DAF-FM-DA. (**C**) The S-nitrosothiol content was determined by the modified Saville method. (**D**) Changes of ONOO^−^ levels in the root apical parts were monitored by microscopy using 20 μM APF. Effects of RNS modulators on physiological parameters were determined as (**E**) root lengths and (**F**) root fresh weights. The data are presented as means ± SD (A–D: *n* ≥ 3; E–F: *n* = 30) relative to the value of tested parameters measured for control plants grown in media without ROS modulators. Significantly different means from control values are denoted by asterisks (ANOVA, * *p* < 0.05, ** *p* < 0.01, *** *p* < 0.001).

To test the involvement of NO in plant root development, GSNO was added to the growth medium as an NO donor and S-nitrosating compound. Significantly higher levels of NO and ONOO^−^ in the roots were observed in plants of both genotypes grown in media containing 100 μM GSNO (Figure 2B,D). GSNO effects on NO and ONOO^−^ levels were not influenced by simultaneous N6022 addition. GSNOR activity was not significantly influenced by GSNO application in both genotypes, whereas the combination of GSNO and N6022 reduced GSNOR activity (Figure 2A). GSNO strongly induced S-nitrosothiol levels in both genotypes and this effect was further increased in the case of simultaneous media supplementation with GSNO and N6022, mainly in *S. lycopersicum* cv. Amateur (Figure 2C). GSNO in the growth medium had a slight positive effect on root growth in *S. lycopersicum* cv. Amateur, which was suppressed in the presence of GSNOR inhibitor. GSNO did not influence the root weight, but a significant positive effect was detected in the presence of N6022 (Figure 2E,F).

Application of NO scavenger PTIO to the growth medium had a positive effect on NO and ONOO^−^ levels and GSNOR activity in 9-day seedlings of both *Solanum* spp. genotypes (Figure 2A,B,D), whereas no changes in S-nitrosothiol levels were detected (Figure 2C). Simultaneous application of PTIO and N6022 eliminated the positive effect of PTIO on GSNOR activity and in contrast, increased S-nitrosothiol levels. PTIO had a strong inhibitory effect on the root growth; however, a positive effect was recorded for the root weight. Interestingly, the simultaneous application of N6022 did not influence the effects of PTIO on root growth (Figure 2E,F). Addition of tested RNS modulators to the growth media stimulated protein nitration in the roots of both genotypes, where the observed intensity of nitration was significantly higher in *S. lycopersicum* cv. Amateur (Supplementary Figure S2). Collectively, these results underscore the importance of GSNOR activity in the control of root growth in *Solanum* spp. mediated by regulation of both NO and S-nitrosothiols levels in the root tissues.

### 3.4. Activities of Enzymes of ROS Metabolism in the Roots Under Non-Stress Conditions are Regulated by GSNOR Through Protein S-Nitrosation

**Figure 3:**
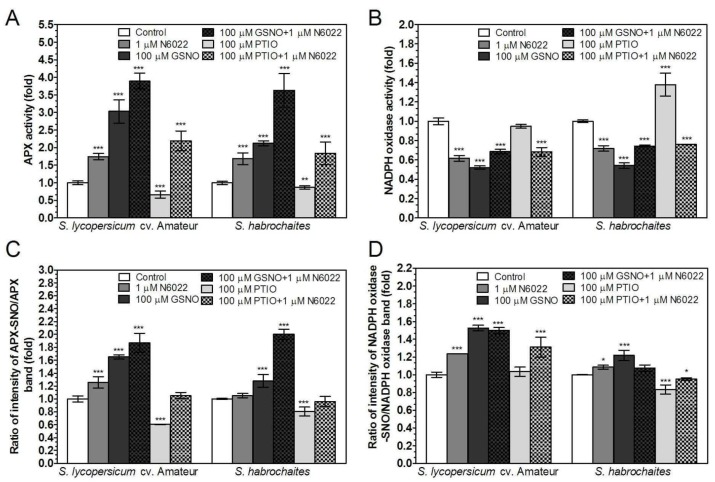
Effects of RNS modulators on APX and NADPH oxidase activity and S-nitrosation status in tomato roots. (**A**) APX activity was evaluated spectrophotometrically at 25 °C by monitoring the consumption of H_2_O_2_ at λ = 2 90 nm. (**B**) NADPH oxidase-catalysed O_2_^−^ generation in purified membrane fractions from tomato roots was determined spectrophotometrically at 25 °C by reduction of the tetrazolium salt XTT. S-nitrosation status for (**C**) APX and (**D**) NADPH-oxidase was determined as the ratio of intensities of detected S-nitrosylated protein vs. total protein by the Western blot method quantified using ImageJ 1.33 software (Supplementary Figure S3). The data are presented as means ± SD (*n* ≥ 3). Significantly different means from the control values are denoted by asterisks (ANOVA, * *p* < 0.05, ** *p* < 0.01, *** *p* < 0.001).

In order to gain additional insights into the interrelations of NO-dependent signaling with ROS pathways mediated through S-nitrosation, ascorbate peroxidase (APX) and NADPH oxidase as important enzymes involved in the metabolism and control of ROS in plant growth and responses to abiotic stress stimuli were selected in this study. APX activity was significantly increased in the roots of both genotypes grown in medium supplemented with N6022 or GSNO, with stronger effects of GSNO found in presence of N6022 (Figure 3A). Application of PTIO had an inhibitory effect on APX activity in both tomato genotypes, whereas APX activity increased after PTIO application in combination with N6022. About 40% lower NADPH oxidase activity was observed after application of N6022 or GSNO in both tomato genotypes, whereas NADPH oxidase activity was increased by PTIO in *S. habrochaites*, but not in *S. lycopersicum* cv. Amateur. Interestingly, GSNOR inhibitor N6022 eliminated the stimulatory effect of PTIO, and decreased NADPH oxidase activity was observed in both genotypes (Figure 3B).

Additionally, to test whether APX and NADPH oxidase activities were modulated by S-nitrosation, we quantified APX and NADPH protein in S-nitrosated protein fraction purified by the biotin-switch method. Increased S-nitrosation of APX and NADPH oxidase was observed mainly in *S. lycopersicum* cv. Amateur after application of N6022 or GSNO; moreover, simultaneous application of N6022 with GSNO strongly potentiated APX S-nitrosation in *S. habrochaites*. PTIO application significantly decreased S-nitrosation of APX in both genotypes, whereas decreased S-nitrosation of NADPH oxidase by PTIO was detected only in *S. habrochaites* (Figure 3C, Supplementary Figure S3). Therefore GSNOR, in its function of the master regulator of cellular protein S-nitrosation, is involved in the control of ROS metabolism mediated by S-nitrosative modifications of APX and NADPH oxidase.

### 3.5. GSNOR is Differentially Involved in Root Responses of Solanum spp. Genotypes to Salinity and Heavy Metal Stress

**Figure 4:**
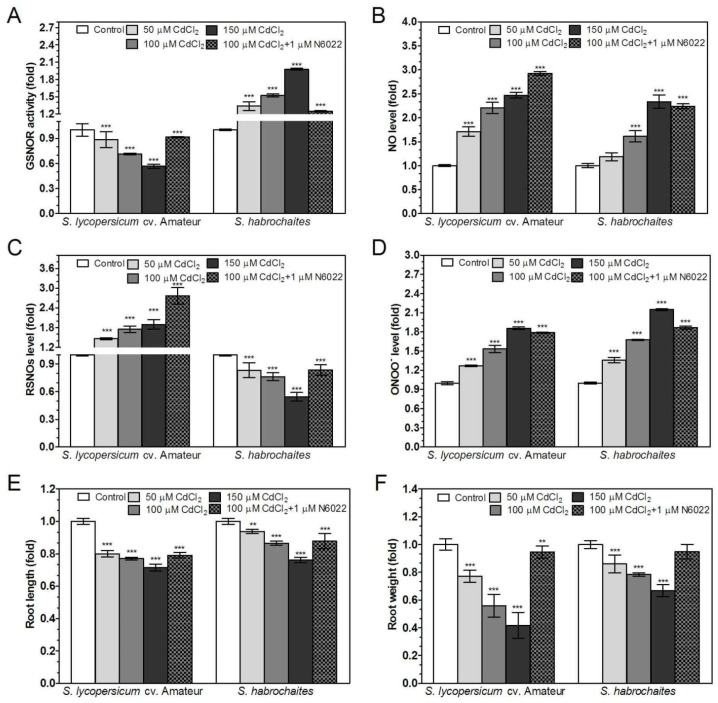
Effects of cadmium exposure on GSNOR activity, RNS levels and root growth in two *Solanum* spp. genotypes. Plants were grown in the growth medium containing 50, 100, 150 μM CdCl_2_ or combination of 100 μM CdCl_2_ with 1 μM GSNOR inhibitor N6022. (**A**) GSNOR activity was evaluated spectrophotometrically at 25 °C by monitoring the decrease of NADH absorbance at 340 nm. (**B**) Changes in NO levels in the root apical parts of were monitored by confocal microscopy using 20 μM DAF-FM-DA. (**C**) The S-nitrosothiol content was determined by the modified Saville method. (**D**) Changes in ONOO^−^ levels in the root apical parts of were monitored by confocal microscopy using 20 μM APF. (**E**,**F**) The effect of cadmium stress on plant growth was determined as (**E**) root lengths and (**F**) root fresh weights. As negative controls for NO and ONOO^−^ determination, roots were pre-incubated with 200 μM NO scavenger cPTIO or 20 μM ONOO^−^ scavenger ebselen, respectively, which completely abolished the fluorescence signal (data not shown). The data are presented as means ± SD (A–D: *n* ≥ 3; E–F: *n* = 30) relative to the value of the tested parameter measured for control plants grown in media without cadmium. Significantly different means from the control values are denoted by asterisks (ANOVA, * *p* < 0.05, ** *p* < 0.01, *** *p* < 0.001).

**Figure 5:**
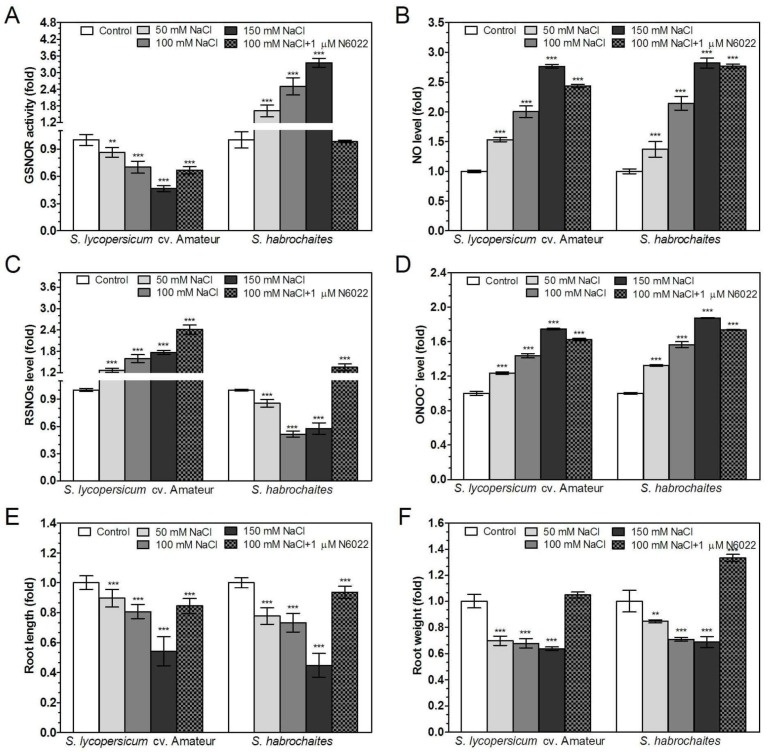
Effect of salinity stress on GSNOR activity, RNS levels and root growth in two *Solanum* spp. genotypes. Plants were grown in the growth medium containing 50, 100, 150 mM NaCl or combination of 100 mM NaCl with 1 μM N6022. (**A**) GSNOR activity was evaluated spectrophotometrically at 25 °C by monitoring the decrease of NADH absorbance at 340 nm. (**B**) Changes in NO levels in the root apical parts were monitored by confocal microscopy using 20 μM DAF-FM-DA. (**C**) The S-nitrosothiol content was determined by the modified Saville method. (**D**) Changes in ONOO^−^ levels in the root apical parts of roots were monitored by confocal microscopy using 20 μM APF. Effect of salinity stress on plant growth was evaluated as (**E**) root lengths and (**F**) root fresh weights. As negative controls for NO and ONOO^−^ determination, roots were pre-incubated with 200 μM NO scavenger cPTIO or 20 μM ONOO^−^ scavenger ebselen, respectively, which completely abolished the fluorescence signal (data not shown). The data are presented as means ± SD (A–D: *n* ≥ 3; E–F: *n* = 30) relative to the value of control plants grown without NaCl. Significantly different means from the control values are denoted by asterisks (ANOVA, * *p* < 0.05, ** *p* < 0.01, *** *p* < 0.001).

Abiotic stress responses were studied in 9-day *Solanum* spp. seedlings exposed to salinity (media supplemented with 50, 100 or 150 mM NaCl) or heavy metal stress (media supplemented with 50, 100 or 150 μM CdCl_2_). The involvement of GSNOR in plant responses to abiotic stress conditions (100 mM NaCl or 100 μM CdCl_2_) was further tested by simultaneous addition of 1 μM N6022. In both tomato genotypes, the inhibitory effects of increasing NaCl and CdCl_2_ concentrations on root growth and root weight were observed (Figure 4E,F and Figure 5E,F). N6022 suppressed negative effects of both types of abiotic stress only in case of the root weight parameter. Both abiotic stress stimuli increased NO and ONOO^−^ levels in the root apical parts in a concentration-dependent manner. Moreover, N6022 increased NO and ONOO^−^ levels in plant roots exposed to both types of abiotic stress (Figure 4B,D and Figure 5B,D).

Significant differences between studied tomato genotypes were recorded in stress-induced modulations of GSNOR activities and S-nitrosothiol levels. In the *S. habrochaites* genotype, GSNOR activity increased in response to increased salt and cadmium concentrations in the medium (Figure 4A and Figure 5A). In contrast, for *S. lycopersicum* cv. Amateur, decreased GSNOR activities were recorded after exposure to both stress conditions. Application of N6022 partially eliminated the effect of both abiotic stresses to GSNOR activity, except for *S. lycopersicum* cv. Amateur under salinity stress. Abiotic stress stimuli triggered increased S-nitrosothiol levels in *S. lycopersicum* cv. Amateur; on the contrary, decreased S-nitrosothiol levels were found in *S. habrochaites* during both types of abiotic stress (Figure 4C and Figure 5C). N6022 treatment further increased S-nitrosothiol levels during both types of stress in *S. lycopersicum* cv. Amateur but only under salinity stress in *S. habrochaites.*

### 3.6. APX and NADPH Oxidase Are Differentially Modulated by Abiotic Stress Conditions in Solanum spp. Genotypes

**Figure 6:**
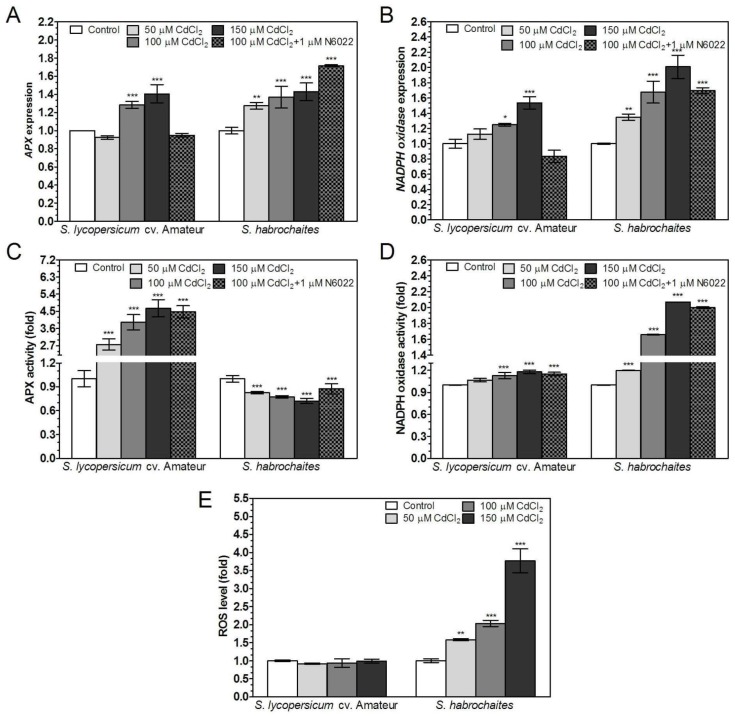
APX and NADPH oxidase gene expression, enzyme activity and ROS levels in two *Solanum* spp. genotypes exposed to cadmium stress. Relative *APX* and *NADPH oxidase* gene expression in plant roots exposed to 50, 100, 150 μM CdCl_2_ or combination of 100 μM CdCl_2_ with 1 μM N6022 was determined by qPCR. (**A**) *APX* and (**B**) *NADPH oxidase* expression levels were normalized to *GAPDH* and *EF1α* mRNA levels. (**C**) APX activity was evaluated spectrophotometrically at 25 °C by monitoring the consumption of H_2_O_2_ at λ = 290 nm. (**D**) NADPH oxidase-catalysed O_2_^−^ generation in purified membrane fractions from tomato roots was determined spectrophotometrically at 25 °C by reduction of the tetrazolium salt XTT. (**E**) Quantification of detected ROS levels in the root apical parts by confocal microscopy using 20 μM H_2_DCF DA (Supplementary Figure S7) was performed by ImageJ 1.33 software. The data are presented as means ± SD (*n* ≥ 3) relative to the gene expression levels or signal intensities measured for control grown without cadmium. Significantly different means from the control are denoted by asterisks (ANOVA, * *p* < 0.05, ** *p* < 0.01, *** *p* < 0.001).

**Figure 7:**
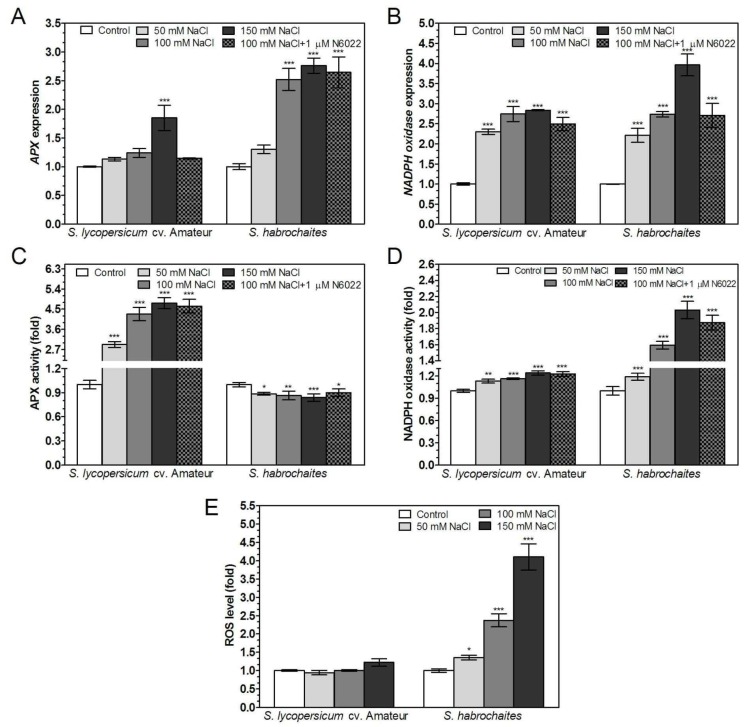
APX and NADPH oxidase gene expression, enzyme activity and ROS levels in two *Solanum* spp. genotypes exposed to salinity stress. Relative *APX* and *NADPH oxidase* gene expression during exposure to 50, 100, 150 mM NaCl or combination of 100 mM NaCl with 1 μM N6022 was determined by qPCR. (**A**) *APX* and (**B**) *NADPH oxidase* expression were normalized to *GAPDH* and *EF1α* mRNA levels. (**C**) APX activity was evaluated spectrophotometrically at 25 °C by monitoring the consumption of H_2_O_2_ at λ = 290 nm. (**D**) NADPH oxidase-catalysed O_2_^−^ generation in purified membrane fractions of tomato roots was determined spectrophotometrically at 25 °C by reduction of the tetrazolium salt XTT. (**E**) Quantification of detected ROS levels in root apical parts by confocal microscopy using 20 μM H_2_DCF DA was performed by ImageJ 1.33 software. The data are presented as means ± SD (*n* ≥ 3) relative to the gene expression level/signal intensity measured for unstressed control, given as 1. Significantly different means from the control are denoted by asterisks (ANOVA, * *p* < 0.05, ** *p* < 0.01, *** *p* < 0.001).

Abiotic stress led to differential responses associated with increased ROS levels in wild genotype *S. habrochaites* and in cultivated genotype *S. lycopersicum cv.* Amateur. Expression of *APX* and *NADPH oxidase* was significantly increased with increasing concentrations of NaCl or CdCl_2_ in the medium in both genotypes (Figure 6A,B and Figure 7A,B). Application of the GSNOR inhibitor N6022 did not influence the expression of *APX* or *NADPH oxidase* in case of salinity stress (Figure 7A,B), whereas under cadmium stress N6022 caused *APX* and *NADPH oxidase* down-regulation in *S. lycopersicum* cv. Amateur and in contrast, induced expression of *NADPH oxidase* in *S. habrochaites* (Figure 6A,B). APX activity increased up to 5 times with increasing concentration of NaCl or CdCl_2_ in *S. lycopersicum* cv. Amateur, conversely, slightly decreased APX activity was recorded in *S. habrochaites* (Figure 6C and Figure 7C). A strong increase of NADPH oxidase activity during stress conditions in *S. habrochaites* contrasted with minimal changes in *S. lycopersicum* cv. Amateur (Figure 6D and Figure 7D). An N6022 effect on the activity of both enzymes was recorded for NADPH oxidase activity in *S. habrochaites* (Figure 6D, Figure 7D). In accordance with detected changes in activities of key enzymes of ROS metabolism in studied tomato genotypes, ROS levels were significantly increased by salinity and cadmium stress in *S. habrochaites* only (Figure 6E, Figure 7E, Supplementary Figure S4).

**Figure 8:**
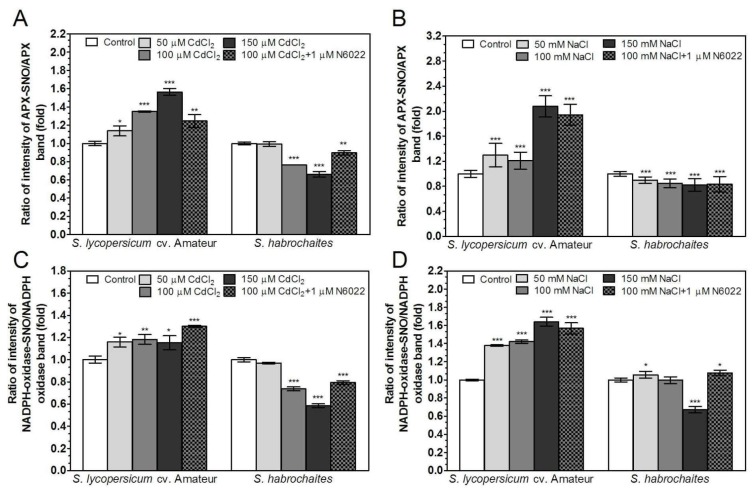
Effects of cadmium and salinity stress on S-nitrosation status of APX and NADPH oxidase in tomato roots. S-nitrosation status of (**A**,**B**) APX and (**C**,**D**) NADPH-oxidase in plant exposed to cadmium (**A**,**C**) or NaCl (**B**,**D**) was calculated as ratios of band intensities of S-nitrosated protein (APX-SNO and NADPH-oxidase-SNO, respectively) vs. band intensities of total APX or NADPH oxidase protein determined by Western blot method using ImageJ 1.33 software (Supplementary Figures S5 and S6). Values indicate relative changes of each immunoreactive band compared to the control plants, to which the value of 1 was assigned. The data are presented as means ± SD (*n* ≥ 3). Significantly different means from the control values are denoted by asterisks (ANOVA, * *p* < 0.05, ** *p* < 0.01, *** *p* < 0.001).

Based on previous reports [10,13,15], we also addressed the role of cysteine S-nitrosation in the regulation of APX and NADPH oxidase activity in tomato roots under abiotic stress conditions. Significant differences were found in measured intensities of S-nitrosation between studied *Solanum* spp genotypes. Increased S-nitrosation of APX and NADPH oxidase was detected only in *S. lycopersicum* cv. Amateur, in contrast to decreased S-nitrosation in *S. habrochaites* (Figure 8, Supplementary Figures S5 and S6). Besides observed differences in S-nitrosation, we detected also intensive modulations of protein nitration in salinity- or cadmium-stressed plant roots in both studied genotypes (Supplementary Figure S7).

## 4. Discussion

Important roles of RNS and ROS in plant root growth, development and stress responses have been previously widely demonstrated [13,46,47,48,49,50]. Furthermore, a key role of GSNOR as a master regulator of protein S-nitrosation in the maintenance of S-nitrosothiols homeostasis during plant development has been recognized (reviewed in [24]). In *A. thaliana* mutant plants, loss of GSNOR function causes developmental defects such as suppression of apical dominance, reduced primary root, inhibition of lateral root formation, reduction of hypocotyl or reduced fertility [18,21,51,52,53]. To study the involvement of GSNOR in regulatory mechanisms during tomato root growth, we exploited a pharmacological approach using synthetic GSNOR inhibitor N6022 with proven inhibitory effects on plant GSNOR [22,50]. In our study, application of the GSNOR inhibitor N6022 to agar growth medium only partially blocked GSNOR activity, which was transduced into strongly increased S-nitrosothiol levels, slightly increased RNS levels and stimulated root growth. This is in agreement with a previous report, where an N6022 treatment markedly suppressed GSNOR activity and increased S-nitrosothiols levels in poplar leaves exposed to 3-day chilling treatment [11].

GSNO is regarded as a reservoir of NO in plant cells and a potential physiological donor of NO; moreover, GSNO can act as a trans-nitrosylation agent able to transfer an NO-group to protein thiols [54,55]. Fernández-Marcos et al. [48] used various NO donors (SNAP, SNP, GSNO) to show that high levels of NO inhibit the growth of *Arabidopsis* primary roots and that the extent of inhibition is dependent on used concentrations of NO donors. Compared to other tested NO donors, 1 mM GSNO concentration had the lowest inhibitory effect. Based on previous studies [54,55], 100 μM GSNO concentrations in the growth media were used in this study. Exogenously added GSNO significantly increased S-nitrosothiol levels in both tomato genotypes, which can have important roles in regulations of metabolic processes including S-nitrosation of the key enzymes of ROS metabolism, APX and NADPH oxidase. The positive effect of NO donor SNP on root growth was previously demonstrated in tomato [46] but not in *Lupinus luteus* [56]. Interestingly, NO donor SNP applied in 100 and 250 μM concentrations restored root growth inhibition induced by iron deficiency in *Arachis hypogaea*, whereas 500 μM SNP showed inhibitory effects [57].

Primary tomato root growth was significantly inhibited in the presence of NO scavenger PTIO in the growth medium, in agreement with previous reports on tomato and other species [46,48,55,58]. Cheng et al. [11] reported that chilling treatment for 3 days and pre-treatment with 30 μM cPTIO for 12 h markedly increased GSNOR activity in poplar leaves. PTIO reacts with NO to form NO_2_^·^ radical; however, even at millimolar concentrations PTIO or its derivative cPTIO cannot prevent a partial conversion of NO to ONOO^−^ in its fast reaction with superoxide [59]. Superoxide accumulates in dividing root cells and expanding meristem tissue, while H_2_O_2_ is localized namely in the root elongation zone [60]. Significantly increased ONOO^−^ levels were detected in root tips of studied tomato genotypes, suggesting possible inhibitory effects of increased ONOO^−^ concentrations to enzyme activities through nitration action of ONOO^−^. Application of NO scavenger PTIO resulted in decreased RNS levels and further disturbance in RNS homeostasis which can lead to activation of plant stress responses [61,62,63,64]. We detected significantly increased RNS levels in 9-day seedlings after PTIO application but with different intensities among studied genotypes. Stronger growth inhibition after PTIO application associated with higher levels of ONOO^−^ and higher GSNOR activity were observed in *S. habrochaites* in comparison to *S. lycopersicum* cv. Amateur.

NO levels in the initial stage of seedlings growth can significantly affect ROS levels, necessary for plant growth and development but also potentially causing oxidative stress leading to inhibited plant development [6,65,66]. NO and GSNO can participate in S-nitrosation or trans-nitrosylation reactions regulating activities of enzymes involved in ROS production or degradation: NADPH oxidase is inhibited whereas APX activated by S-nitrosation; however, APX can be inhibited by tyrosine nitration [2,14,15,67]. Regulation of plant NADPH oxidase (AtRBOHD) activity by reversible S-nitrosation has been observed in vitro and in vivo during Pseudomonas syringae infection in A. thaliana [4]. Initially, inhibition of APX activity by increasing concentrations of GSNO was described in tobacco leaves [68]. However, recent studies showed an opposite effect, as different APX isozymes treated with NO donor showed increased activity in the root nodules of soybean [69]. Similarly, in seeds of *Anticaria toxicaria* treated with gaseous NO, an APX S-nitrosation and enhanced activity was described, contributing to increased tolerance to seed desiccation [70]. Begara-Morales et al. [10] showed that GSNO enhanced pea APX activity through S-nitrosation at Cys32, located near the propionate side chain of the enzyme haem group [71,72]. In our experiment, increased S-nitrosation in both tomato genotypes after application of N6022 and GSNO corresponded with increased levels of protein S-nitrosothiols, increased APX activity and decreased NADPH oxidase activity. S-nitrosation of APX and NADPH oxidase in both genotypes was significantly decreased or increased after application of NO scavenger PTIO or GSNO in combination with N6022, respectively. Taken together, our results confirm the central role of GSNOR in the regulation of S-nitrosation under non-stress conditions in tomato roots, which include the control of S-nitrosation status of key enzymes of ROS metabolism.

ROS and RNS signaling pathways are known to form a coordinated network that regulates plant responses to environmental stimuli; however, only a limited number of studies addressed the involvement of RNS and S-nitrosation in regulations of key enzymes of ROS metabolism during abiotic stress in plants [66]. Increased NO production during salinity stress was observed in tobacco cell suspension, Arabidopsis and sunflower roots, and citrus, maize and olive leaves [20,70,73,74,75,76,77,78]. Salinity stress activates ROS production, resulting in oxidative stress and subsequent activation of antioxidant enzymes depending on plant sensitivity/tolerance to salinity [58,79]. APX has been demonstrated as a key enzyme of the ascorbate-glutathione cycle is crucial for the tolerance to salinity stress observed in lentils [79], citrus plants [80], or potato callus [81]. Camejo et al. [25] reported that long-term salinity stress 150 mM NaCl increased GSNOR activity and NO production in pea mitochondria, whereas Manai et al. [82] observed that externally applied NO donor under salinity stress increased activities of antioxidant enzymes in the roots of *S. lycopersicum*. Enhancement of the APX activity by S-nitrosation together with increased levels of NO, S-nitrosothiols was reported in pea leaves exposed to salinity stress [10]. Several reports have provided evidence for a rapid increase in NO production under heavy metals stress [83,84,85]. In soybean cell culture, the ability of NO to act through activation of antioxidant enzymes was observed after application of Cu^2+^ and Cd^2+^ [86]. Similarly, exogenous NO alleviated the toxicity of arsenic in rice and mung beans through induction of antioxidant enzymes [87,88,89].

**Figure 9:**
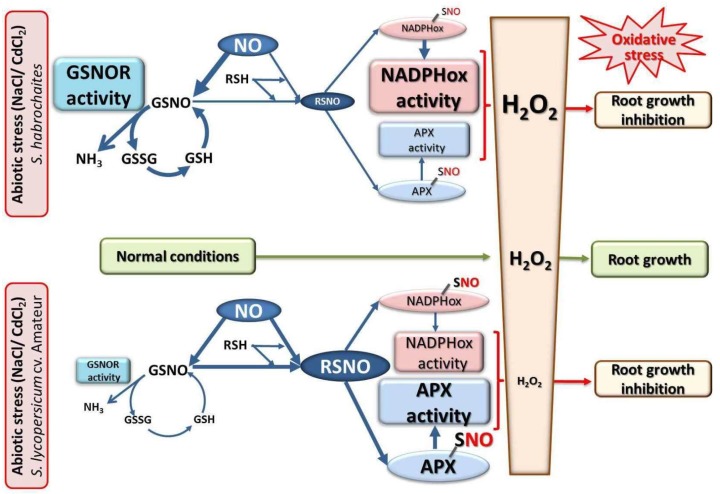
Regulation of *Solanum* spp. root defence responses to abiotic stress conditions by S-nitrosation. S-nitrosation serves as a regulatory mechanism affecting activities of enzymes involved in the metabolism of ROS, namely, H_2_O_2_, in tomato responses to long-term exposure to salinity or cadmium stress. Stress-induced increase in GSNOR activity and subsequent decrease of S-nitrosothiol levels in *S. habrochaites* results in reduced S-nitrosation status of APX and NADPH oxidase, associated with decreased APX and increased NADPHox activities, elevated H_2_O_2_ levels and oxidative stress conditions in the root cells. In contrast, stress-triggered down-regulation of GSNOR activity in *S. lycopersicum* cv. Amateur leads to increased S-nitrosothiol levels and increased S-nitrosation protein status, which has an inhibitory effect on NADPHox activity and *vice versa*, increased APX activity, resulting in lower ROS levels in roots cells. In both genotypes, disturbed ROS homeostasis under stress conditions may contribute to observed inhibition of the root growth and development.

In our study, we analyzed two *Solanum* spp. genotypes *S. habrochaites* and *S. lycopersicum* cv. Amateur, differing in previously characterized degree of resistance to biotic stress [31,33,90]. We found that increased salinity and cadmium concentrations had similar effects on the inhibition of root growth and development in both genotypes, with *S. habrochaites* showing lower responses to highest concentrations of NaCl or CdCl2. We showed different modulations of their responses during abiotic stress at the level of enzymes involved in ROS metabolism. It has been proposed that S-nitrosation can regulate H_2_O_2_ levels by controlling both the ROS producing and antioxidant enzymes [91]. We found that responses to salinity and cadmium were mediated by activation of different mechanisms in studied genotypes (Figure 9). Abiotic stress resulted in a significant increase of S-nitrosothiol levels and APX activity in *S. lycopersicum* cv. Amateur. On the contrary, changes in NADPH oxidase activity and production of ROS were less significant. In *S. habrochaites* decreased S-nitrosothiols level and APX activity were detected, whereas increased NADPH oxidase activity, leading to increased ROS levels, was observed. The ability to regulate activities of APX and NADPH oxidase by S-nitrosation in response to abiotic stress was also confirmed in both tomato genotypes.

The level of S-nitrosation is indirectly regulated by GSNOR activity [17] which is modulated in response to abiotic stress stimuli in a number of model plants. Barroso et al. [92] described a 30% reduction of GSNOR activity and expression in pea leaves after treatment with 50 μM cadmium chloride. A similar trend was observed in Arabidopsis seedlings grown in presence of 500 μM arsenic [93]. In the roots, stem and leaves of the resistant genotype of sugar melon (*C. melo*), an increased GSNOR activity was detected 24 h after exposure to heat stress at 42 °C, whereas GSNOR was slightly reduced in the susceptible genotype *C. sativus* cv. Stela [41]. Long-term cold stress of poplar (*Populus yunnanensis* Dode) leaves at 4 °C caused decreased GSNOR activity, increased S-nitrosothiols and increased activities of the antioxidant ascorbate-glutathione cycle enzymes [11]. Exposure of pea roots to 50 μM CdCl_2_ for 15 days decreased NO levels and increased ROS produced by NADPH oxidase [94]. Six abiotic stress factors (heat and cold stress, drought, mechanical injury, continuous light and darkness) significantly increased GSNOR activity and modulated S-nitrosothiol levels in pea leaves [95]. In our experiment, responses of *Solanum* spp. genotypes during long-term abiotic stress were significantly different in changes of GSNOR activity and modulation of protein S-nitrosothiol levels, as well as in activities of ROS metabolism enzymes, as discussed above. In consequence, the modulation of APX and NADH oxidase activities resulted in decreased or increased ROS levels in *S. lycopersicum* and *S. habrochaites*, respectively, leading to the root growth inhibition in both genotypes (Supplementary Figure S7, Figure 9).

## 5. Conclusions

In summary, the results of the present study point to the key role of GSNOR in molecular mechanisms underlying the capacity of *Solanum* spp. plants to respond to stress conditions, in agreement with previous studies aimed to characterize roles of ROS and RNS in defense mechanisms of *Solanum* spp. to biotic stress [31,32,33,90]. Therefore, we can assume that observed root growth inhibition in *S. habrochaites* results from increased GSNOR activity by abiotic stress stimuli, and subsequent decrease in S-nitrosothiol levels and decreased S-nitrosation, resulting in activation of NADPH oxidase, deactivation of APX and increased ROS level. Conversely, *S. lycopersicum* cv. Amateur is characterized by elevated S-nitrosation caused by decreased GSNOR activity, stimulating APX activity to detoxify accumulated ROS and to prevent oxidative damage. Decreased ROS levels and disturbances in ROS homeostasis result in root growth inhibition in this genotype. Interestingly, under non-stressed conditions, *S. lycopersicum* cv. Amateur genotypes showed a 2-fold higher level of NO and S-nitrosothiols but also a 2-fold higher GSNOR activity compared to *S. habrochaites*. Collectively, presented data uncover that closely related plant genotypes might respond to abiotic stress stimuli through different modes of modulations in ROS and RNS metabolism, depending on the type of the stress stimulus, its extent and also likely on its duration. Our results stress the high importance of experimental studies on crop plants such as tomato, which might show highly divergent mechanisms of stress responses to those described in model plants like *A. thaliana*. Further experiments are necessary to test the possibility of using parameters of S-nitrosothiol metabolism as a potential predictor of crop tolerance to specific abiotic stress stimuli.

## Supplementary Materials

The following are available online at https://www.mdpi.com/2218-273X/9/9/393/s1, Table S1: Primers used for qPCR analysis, Figure S1: Representative images of 9-day seedlings of Solanum spp. genotypes, Table S2: Comparison of physiological and biochemical parameters in 9-day seedlings of Solanum spp. genotypes, Figure S2: Effects of RNS modulators on protein nitration in Solanum spp. roots, Figure S3: S-nitrosated and total APX and NADPH oxidase protein levels exposed to RNS modulators, Figure S4: Protein nitration in tomato roots exposed to cadmium or salinity, Figure S5: S-nitrosated and total APX and NADPH oxidase protein levels in tomato roots exposed to cadmium, Figure S6: S-nitrosated and total APX and NADPH oxidase protein levels in tomato roots exposed to salinity, Figure S7: ROS levels in roots of Solanum spp. genotypes exposed to cadmium or salinity stress.
