# Supplementary material for: Tomato Root Growth Inhibition by Salinity and Cadmium is Mediated by S-Nitrosative Modifications of ROS Metabolic Enzymes Controlled by S-Nitrosoglutathione Reductase
Source: Biomolecules. 2019 Aug 21;9(9):393. doi: 10.3390/biom9090393 (PMC6788187; doi:10.3390/biom9090393)

Supplementary Material

Jedelská et al. – S-nitrosation in tomato root under abiotic stress

Supplementary Table S1. Primers used for gene expression analysis by qPCR.

Genes	GenBank	Primers	Product length (bp)
EF1α	NM_001247106	fwd: 5′-GGTCATCATCATGAACCATCC-3′<br>rev: 5′-CATACCAGCATCACCGTTCTT-3′	175
GAPDH	U93208	fwd: 5′-AACCGGTGTCTTCACTGACAAGGA-3’<br>rev: 5’-CACCCACAACAAACATGGGAGCAT-3’	110
APX	AY974805	fwd: 5′-GAGGACCTGATGTTCCCTTTC-3′<br>rev: 5′-AAGGTATGGGCACCAGAGAGT-3′	169
NADPH oxidase	AF088276	fwd: 5′-CGGATGGAATGAAGTTGAAAA-3′<br>rev: 5′-AAGCATCAAACAATTCCAACG-3′	206

Supplementary Table S2. Comparison of physiological and biochemical parameters determined in 9-day seedlings of *Solanum* spp. genotypes. Data represent means ± SD (n ≥ 3).

**Parameter**	***S. lycopersicum* cv. Amateur**	***S. habrochaites***
Epicotyl length (cm)	5.05 ± 0.71	2.41 ± 0.27
Root length (cm)	9.65 ± 0.56	7.67 ± 0.43
Root weight (g)	0.18 ± 0.01	0.06 ± 0.003
NO level (rel. u.)	32.24 ± 2.76	19.64 ± 1.28
$ONOO^-$ level (rel. u.)	23.17 ± 3.08	21.01 ± 0.73
ROS level (rel. u.)	6.93 ± 0.13	8.63 ± 0.46
GSNOR activity ($nmol \cdot min^{-1} \cdot g^{-1}$ FW)	11.02 ± 1.28	5.89 ± 0.72
Nitrosothiols level ($nmol \cdot mg^{-1}$ protein)	20.00 ± 1.89	8.09 ± 2.18
APX activity ($\mu mol \cdot min^{-1} \cdot g^{-1}$ FW)	242.29 ± 21.92	224.43 ± 16.12
NADPH oxidase activity ($\mu mol \cdot min^{-1} \cdot g^{-1}$ FW)	87.48 ± 0.12	69.99 ± 0.29
APX expression	1.28 ± 0.04	1.06 ± 0.01
NADPH oxidase expression	0.78 ± 0.03*	1.48 ± 0.02*

* gene expression was normalized to the expression levels of *GAPDH* and *EF1-α*

Supplementary Figure S1. **Representative images of 9-day seedlings of *Solanum* spp. genotypes.** (A) *Solanum lycopersicum* cv. Amateur; (B) *Solanum habrochaites*.

A B

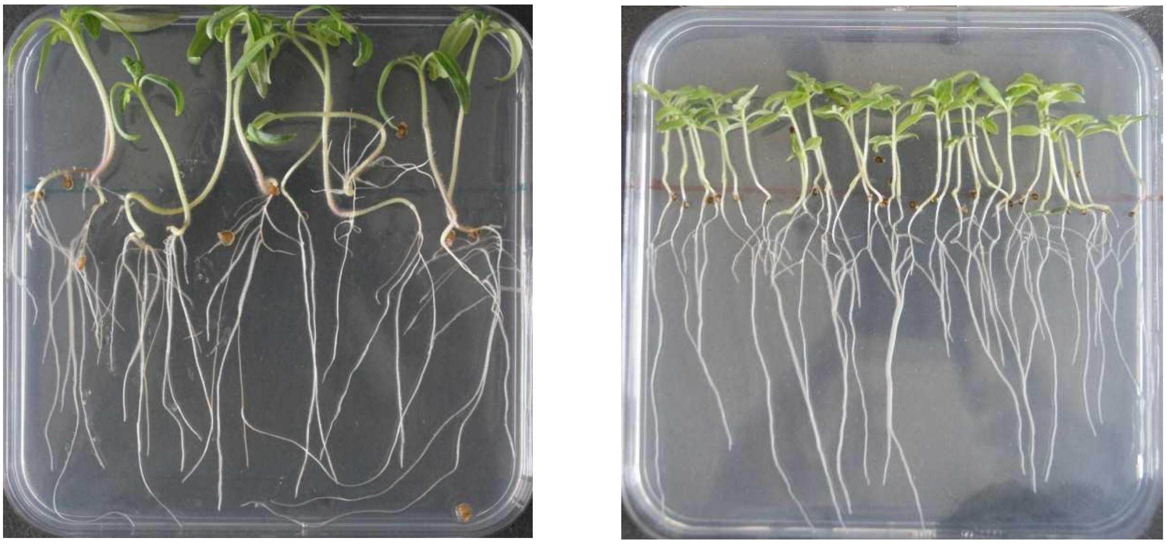

Supplementary Figure S2. Effect of RNS modulators supplementation to the growth media on protein nitration in roots of *Solanum* spp.. Plants of *Solanum* spp. were grown on media supplemented with 1 μM N6022; 100 μM GSNO; 100 μM GSNO + 1 μM N6022; 100 μM PTIO; or 100 μM PTIO + 1 μM N6022. (A) Levels of nitrated proteins were analysed in root extracts (100 μg of protein per lane) by SDS-PAGE and Western blot analysis using a mouse polyclonal antibody against 3-nitrotyrosine. Commercial nitrated BSA (NO_2-BSA, 10 μg), served as the positive control (not shown). (B) Band intensities on Western blot images were analysed using ImageJ 1.33 software. Values indicate a relative increase of each immunoreactive band compared to the control, to which the value of 1 was assigned. N.d., not detected. Data represent means ± SD (n ≥ 3).

A

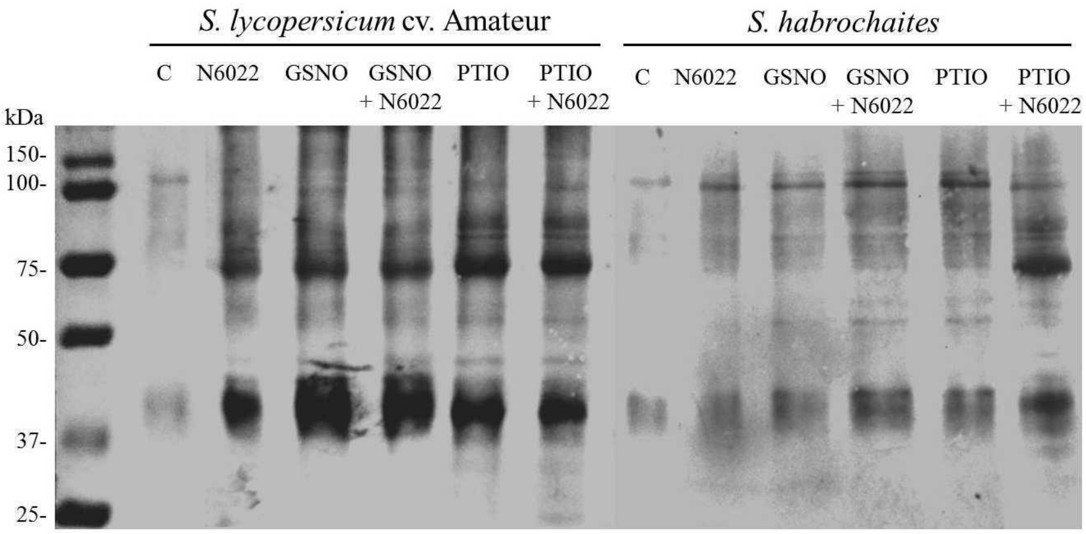

B

S. lycopersicum cv. Amateur					
**Band (kDa)**	**N6022**	**GSNO**	**GSNO +N6022**	**PTIO**	**PTIO +N6022**
**100**	n.d.	n.d.	n.d.	n.d.	n.d.
**75**	2.8 ± 0.06	2.5 ± 0.17	3.5 ± 0.01	2.7 ± 0.01	3 ± 0.30
**40**	3.6 ± 0.05	2.2 ± 0.2	2.8 ± 0.01	4.2 ± 0.30	2.5 ± 0.08

S. habrochaites					
**Band (kDa)**	**N6022**	**GSNO**	**GSNO +N6022**	**PTIO**	**PTIO +N6022**
**100**	1.6 ± 0.05	2.3 ± 0.05	4 ± 0.06	1.9 ± 0.02	5.2 ± 0.40
**75**	1.11 ± 0.02	1.3 ± 0.02	1.2 ± 0.03	3.8 ± 0.20	1.2 ± 0.04
**40**	1.2 ± 0.01	1.1 ± 0.09	1.7 ± 0.03	2.1 ± 0.01	1.4 ± 0.02

Supplementary Figure S3. Detection of S-nitrosated and total APX and NADPH oxidase protein levels in tomato roots exposed to RNS modulators. For the purification of the S-nitrosated proteins from tomato roots, proteins extracted from tomato roots (5 mg) were subjected to the biotin switch method, followed by purification using neutravidin-affinity chromatography. Purified fractions were then analysed by SDS-PAGE (100 μg of protein per lane), followed by Western blotting and immunodetection with anti-APX (1:1000) or anti-NADPH-oxidase (1:1000) antibodies. Detection of total APX and NADPH oxidase protein level was performed by immunoblot analysis using the corresponding antibody with the same dilution as for APX-SNO or NADPH-oxidase-SNO. Roots of tomato genotypes (A) *S. lycopersicum* cv. Amateur, (B) *S. habrochaites* were grown on medium supplemented with 1 μM N6022, 100 μM GSNO, 100 μM PTIO or 1 μM N6022+100 μM GSNO, 1 μM N6022+100 μM PTIO for 9 days. Actin is included as a protein loading control. (C, D) Band intensities were analysed using ImageJ 1.33 software. Values indicate a relative increase of each immunoreactive band compared to the control, to which the value of 1 was assigned. Significantly different means from the control are denoted by asterisks (ANOVA, * $p < 0.05$, ** $p < 0.01$, *** $p < 0.001$).

A

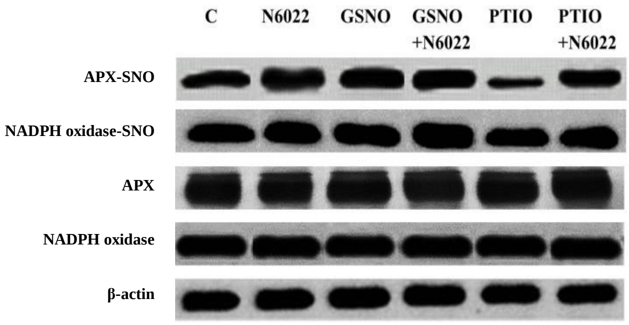

B

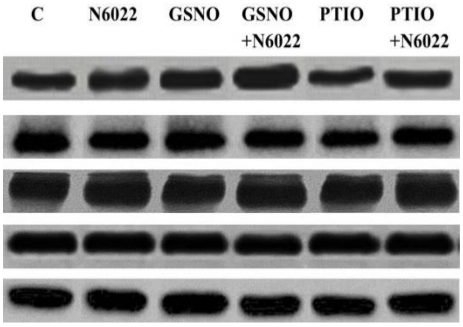

C

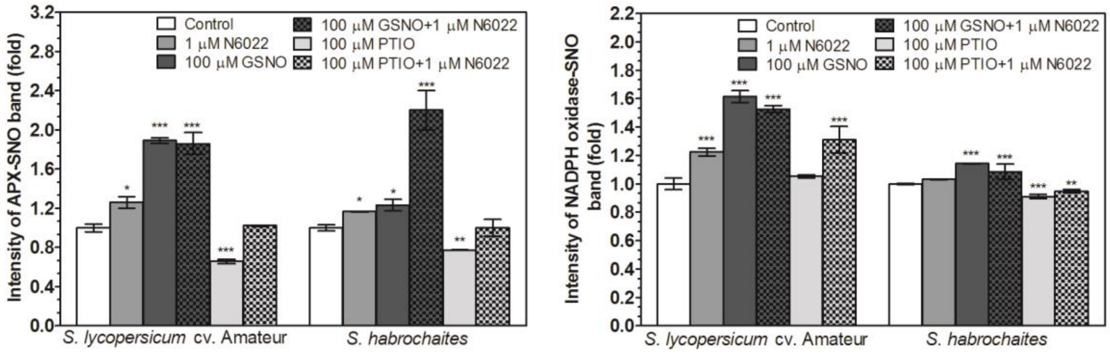

D

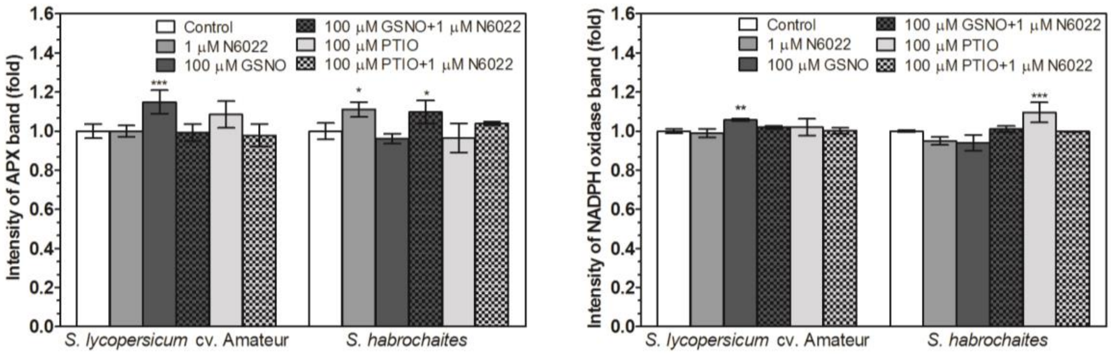

Supplementary Figure S4. Protein nitration in roots of *Solanum* spp. genotypes exposed to cadmium or salinity stress. (A) Levels of nitrated proteins were analysed in root extracts (100 µg of protein per lane) from plants of *Solanum* spp. exposed to 50-100-150 µM $CdCl_2$ and 100 µM $CdCl_2$+1 µM N6022, or 50-100-150 mM NaCl and 100 mM NaCl+1 µM N6022, by SDS-PAGE and Western blot analysis using a mouse polyclonal antibody against 3-nitrotyrosine. Commercial nitrated BSA (NO_2-BSA, 10 µg), served as the positive control (not shown). (B) Band intensities on Western blots were analysed using ImageJ 1.33 software. Values indicate a relative increase of each immunoreactive band compared to the control, to which the value of 1 was assigned. N.d., not detected. Data represent means ± SD (n ≥ 3).

A

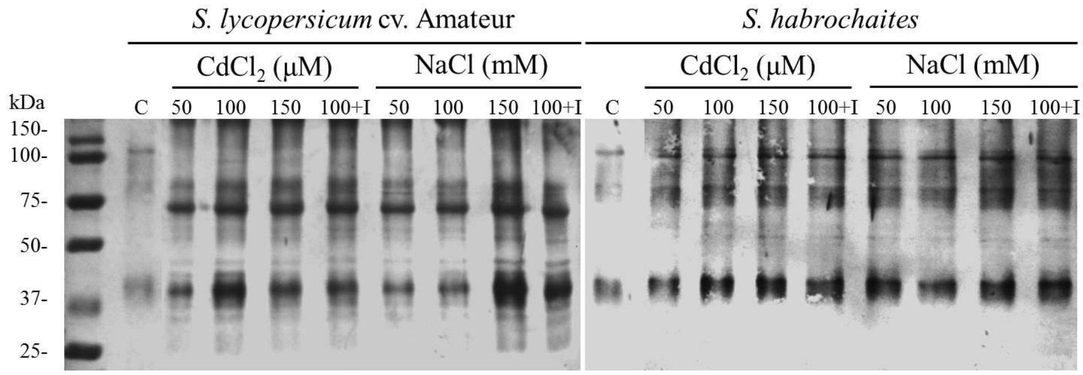

B

				***S. lycopersicum* cv. Amateur**				
		**$CdCl_2$**				**NaCl**		
**Band (kDa)**	**50 µM**	**100 µM**	**150 µM**	**100 µM + 1 µM N6022**	**50 mM**	**100 mM**	**150 mM**	**100 mM + 1 µM N6022**
100	n.d.	n.d.	n.d.	n.d.	n.d.	n.d.	n.d.	n.d.
75	1.9 ± 0.01	2.8 ± 0.3	2.3 ± 0.1	2.8 ± 0.2	1.9 ± 0.05	2.2 ± 0.02	3.2 ± 0.5	2.3 ± 0.05
40	1.3 ± 0.01	2.1 ± 0.08	1.6 ± 0.01	1.6 ± 0.2	1.1 ± 0.07	1.2 ± 0.01	2.6 ± 0.09	2.8 ± 0.2

				S. habrochaites				
		**$CdCl_2$**				**NaCl**		
**Band (kDa)**	**50 µM**	**100 µM**	**150 µM**	**100 µM + 1 µM N6022**	**50 mM**	**100 mM**	**150 mM**	**100 mM + 1 µM N6022**
100	1.6 ± 0.08	2 ± 0.03	2.7 ± 0.12	2.2 ± 0.01	2.7 ± 0.1	2.9 ± 0.01	2.8 ± 0.2	3.1 ± 0.2
75	1.7 ± 0.03	2.1 ± 0.07	2.2 ± 0.06	1.9 ± 0.01	2.3 ± 0.02	1.9 ± 0.04	2.9 ± 0.04	2.7 ± 0.02
40	1.2 ± 0.03	1.6 ± 0.05	1.8 ± 0.1	1.7 ± 0.01	2.1 ± 0.2	1.5 ±0.1	2 ± 0.1	2.2 ± 0.06

Supplementary Figure S5. Detection of S-nitrosated and total APX and NADPH oxidase protein levels in tomato roots exposed to cadmium stress. For the purification of S-nitrosated proteins from tomato roots, extracted proteins (5 mg) were subjected to the biotin switch method, followed by purification using neutravidin-affinity chromatography. Purified fractions were then analysed by SDS-PAGE (100 μg of protein per lane), followed by immunoblotting with anti-APX (1:1000) or anti-NADPH-oxidase (1:1000) antibodies. Detection of total APX and NADPH oxidase protein expression level was performed by immunoblot analysis using corresponding antibodies with the same dilution as for APX-SNO or NADPH-oxidase-SNO. Roots of tomato genotypes (A) *S. lycopersicum* cv. Amateur, (B) *S. habrochaites* were grown on medium supplemented with 50, 100, 150 μM $CdCl_2$ or 100 μM $CdCl_2$+1 μM N6022 for 9 days. Actin was included as a protein loading control. (C, D) Band intensities on Western blots were analysed using ImageJ 1.33 software. Values indicate a relative increase of each immunoreactive band compared to unstressed control, to which the value of 1 was assigned. Significantly different means from the control are denoted by asterisks (ANOVA, * $p < 0.05$, ** $p < 0.01$, *** $p < 0.001$).

A

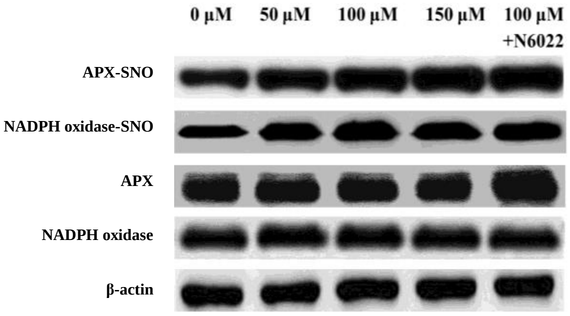

B

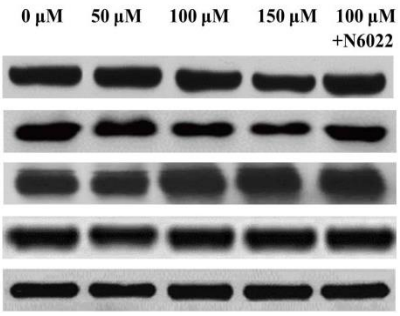

C

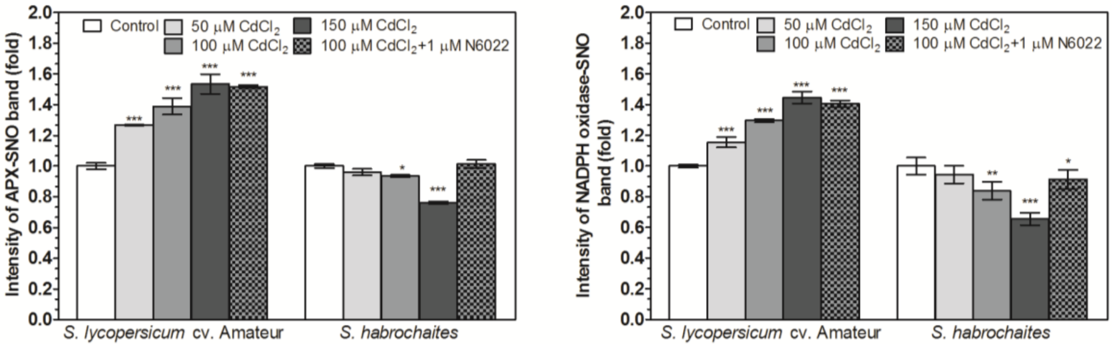

D

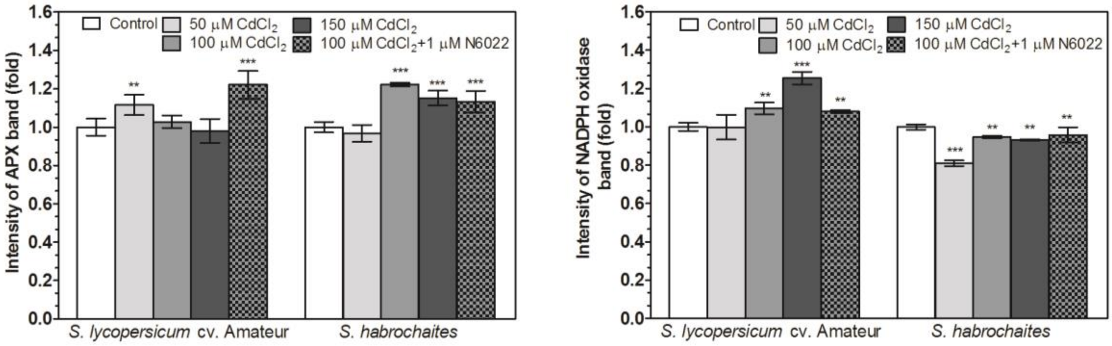

Supplementary Figure S6. Detection of S-nitrosated and total APX and NADPH oxidase protein levels in tomato roots exposed to salinity stress. For the purification of S-nitrosated proteins from tomato roots, extracted proteins (5 mg) were subjected to the biotin switch method, followed by purification using neutravidin affinity chromatography. Purified fractions were then analysed by SDS-PAGE (100 µg of protein per lane), followed by immunoblotting with anti-APX (1:1000) or anti-NADPH-oxidase (1:1000) antibodies. Detection of total APX and NADPH oxidase protein expression level was performed by immunoblot analysis using corresponding antibodies with the same dilution as for APX-SNO or NADPH-oxidase-SNO. Roots of tomato genotypes (A) *S. lycopersicum* cv. Amateur, (B) *S. habrochaites* were grown on medium supplemented with 50, 100, 150 mM NaCl or 100 mM NaCl+1 µM N6022 for 9 days. Actin was included as a protein loading control. (C, D) Band intensities on Western blots were analysed using ImageJ 1.33 software. Values indicate a relative increase of each immunoreactive band compared to the unstressed control, to which the value of 1 was assigned. Significantly different means from the control are denoted by asterisks (ANOVA, * $p < 0.05$, ** $p < 0.01$, *** $p < 0.001$).

A

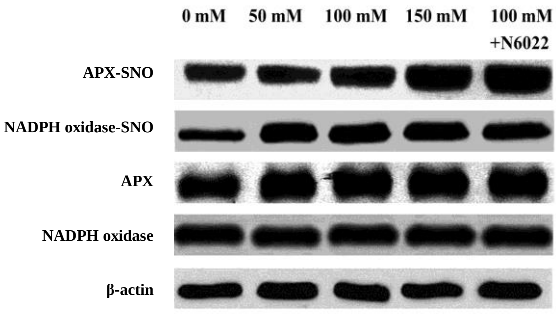

B

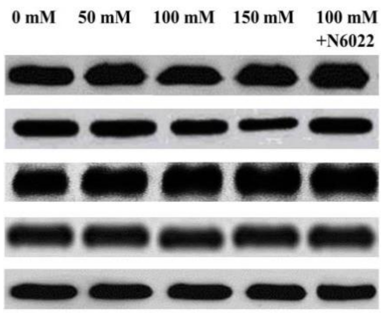

C

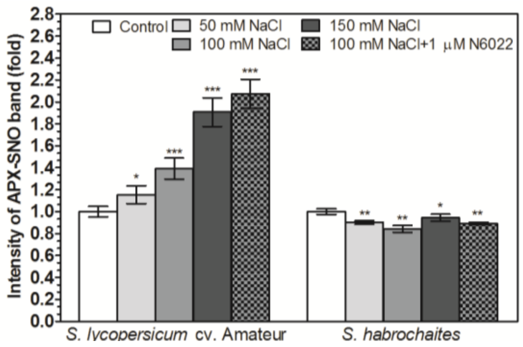

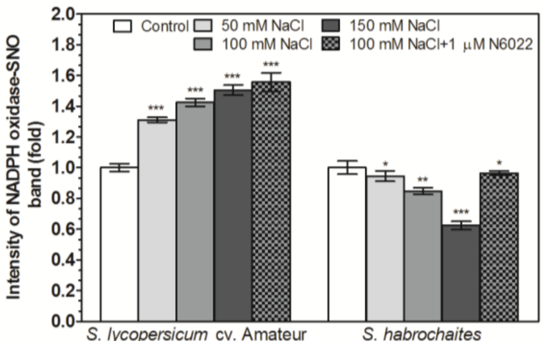

D

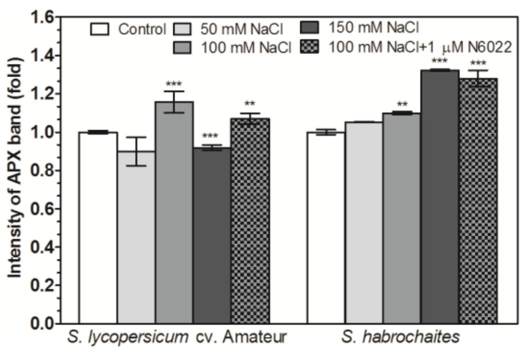

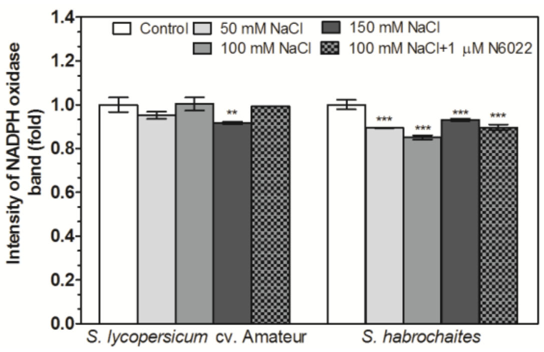

Supplementary Figure S7. ROS levels in roots of *Solanum* spp. genotypes exposed to cadmium or salinity stress. Fluorescent probe H_2DCF DA in 20 µM concentration was used for the detection of ROS in apical parts of roots. Green fluorescence signal detected by fluorescence microscopy corresponds to intracellular ROS levels. As a negative control, roots were incubated with 20 mM ascorbate (images not shown). Scale bar = 200 µm.

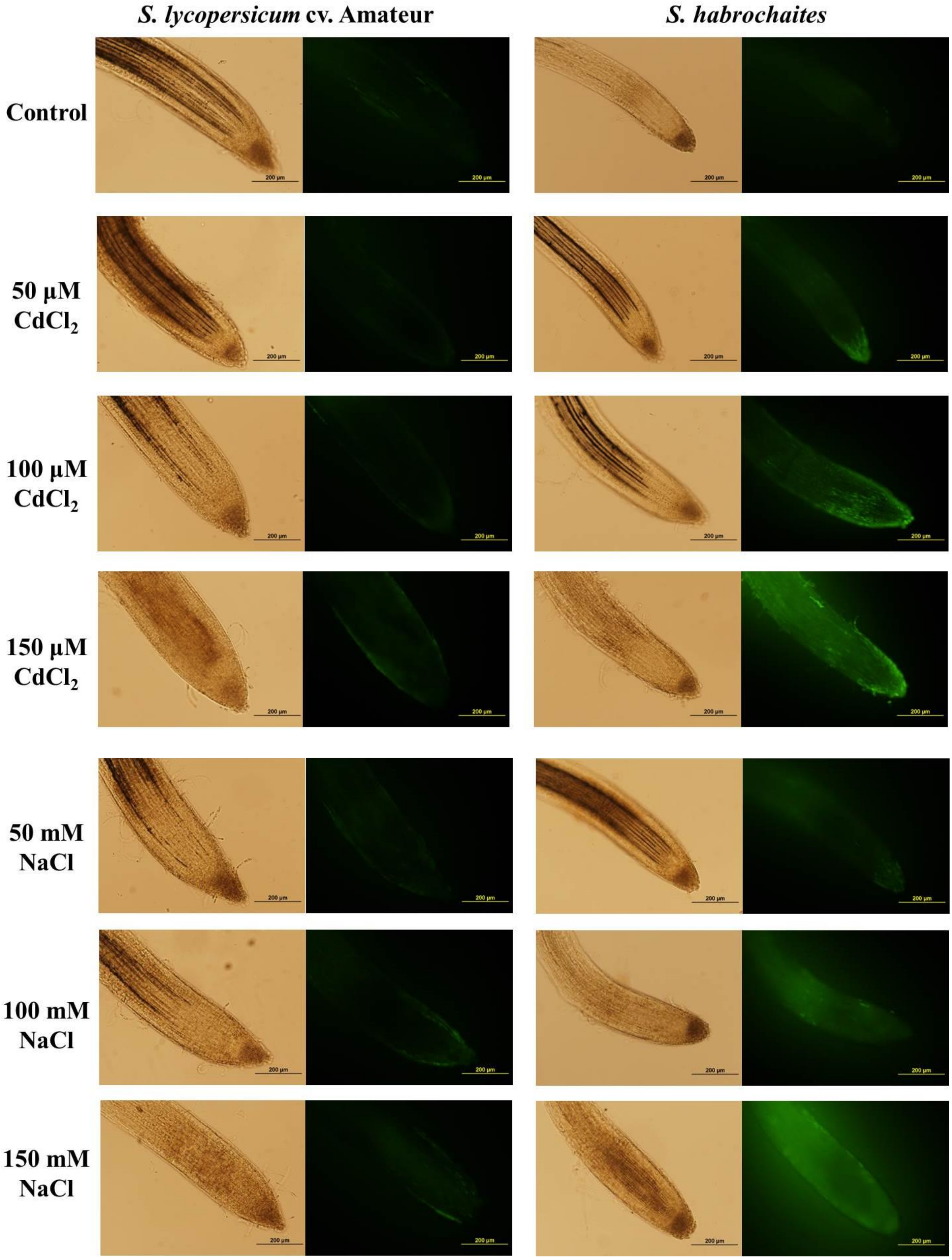

Supplement: Supplementary file 1 [file biomolecules-09-00393-s001.pdf]
